# Implementation of COVID‐19 Preventive Measures and Staff Well‐Being in a Sample of English Schools 2020‐2021

**DOI:** 10.1111/josh.13264

**Published:** 2022-11-30

**Authors:** Neisha Sundaram, Tanya Abramsky, William E Oswald, Sarah Cook, Katherine E Halliday, Patrick Nguipdop‐Djomo, Joanna Sturgess, Georgina Ireland, Shamez N Ladhani, Punam Mangtani, Sinéad M Langan, James R Hargreaves, Chris Bonell, COVID‐19 Schools Infection Survey Study Group

**Affiliations:** ^1^ Department of Public Health, Environments and Society London School of Hygiene & Tropical Medicine WC1H 9SH London UK; ^2^ Department of Global Health and Development London School of Hygiene & Tropical Medicine WC1H 9SH London UK; ^3^ Department of Disease Control London School of Hygiene & Tropical Medicine WC1E 7HT London UK; ^4^ Department of Non‐communicable Disease Epidemiology, London School of Hygiene & Tropical Medicine, London WC1E 7HT; National Heart and Lung Institute, Imperial College London London UK; ^5^ Department of Disease Control London School of Hygiene & Tropical Medicine WC1E 7HT London UK; ^6^ Department of Infectious Disease Epidemiology London School of Hygiene & Tropical Medicine WC1E 7HT London UK; ^7^ Department of Medical Statistics London School of Hygiene & Tropical Medicine WC1E 7HT London UK; ^8^ Public Health Programmes UK Health Security Agency London UK; ^9^ Public Health Programmes, UK Health Security Agency; Paediatric Infectious Diseases Research Group, St George's University of London London UK; ^10^ Department of Infectious Disease Epidemiology London School of Hygiene & Tropical Medicine WC1E 7HT London UK; ^11^ Department of Non‐communicable Disease Epidemiology London School of Hygiene & Tropical Medicine WC1E 7HT London UK; ^12^ Department of Public Health, Environments and Society London School of Hygiene & Tropical Medicine WC1H 9SH London UK; ^13^ Department of Public Health, Environments and Society London School of Hygiene & Tropical Medicine WC1H 9SH London UK

**Keywords:** COVID‐19, schools, burnout, disease control, preventive measures, mental health

## Abstract

**BACKGROUND:**

We examined fidelity and feasibility of implementation of COVID‐19 preventive measures in schools, and explored associations between adherence to these measures and staff well‐being, to inform policy on sustainable implementation and staff wellbeing.

**METHODS:**

Surveys were conducted across 128 schools in England with 107 headteachers and 2698 staff‐members with reference to autumn term 2020, examining school‐level implementation of preventive measures, adherence, and teacher burnout (response rates for headteacher and staff surveys were 84% and 59%, respectively).

**RESULTS:**

The median number of measures implemented in primary and secondary schools was 33 (range 23‐41), and 32 (range 22‐40), respectively; most measures presented challenges. No differences were found regarding number of measures implemented by school‐level socio‐economic disadvantage. High adherence was reported for staff wearing face‐coverings, staff regularly washing their hands, (secondary only) desks facing forwards, and (primary only) increased cleaning of surfaces and student hand‐washing. Adherence to most measures was reported as higher in primary than secondary schools. Over half of school leaders and 42% (517/1234) of other teaching staff suffered from high emotional exhaustion. Higher teacher‐reported school‐wide adherence with measures was consistently associated with lower burnout for leaders and other teaching staff.

**CONCLUSIONS:**

Findings indicate a tremendous effort in implementing preventive measures and an urgent need to support investments in improving teacher wellbeing.

The COVID‐19 pandemic has resulted in over 250 million confirmed COVID‐19 cases and over 5 million deaths worldwide as of November 2021.^1^ Evidence suggests that children are less vulnerable than adults to morbidity and mortality.^2^, ^3^, ^4^, ^5^, ^6^ However, school closures have been widely used as a control measure, resulting in the largest disruption to schooling in recent history.^7^ School closures have adversely impacted students' learning, physical health, mental health and wellbeing, and societal functioning.^8^, ^9^, ^10^, ^11^, ^12^, ^13^, ^14^, ^15^

Faced with the need to minimize such harms while minimizing the role of schools in transmission, many countries reopened schools while implementing various preventive measures.^16^, ^17^, ^18^, ^19^, ^20^ While evidence suggests that schools in the United Kingdom are implementing a large number of preventive measures^16^, ^17^ which are likely to reduce COVID‐19 transmission,^21^ there is little evidence on the fidelity of implementation of different measures, the challenges they raise for schools, or the extent to which students adhere to them.

Mental health has been a concern over the course of the pandemic, including the mental health of children, adolescents, and teachers.^22^, ^23^ Informed by the general literature on teacher mental health and burnout, it is likely that increased workloads and stresses associated with enacting complex preventive measures with limited guidance and support may predispose teachers to burnout.^24^, ^25^, ^26^ Burnout is defined as a psychological syndrome involving emotional exhaustion, depersonalisation and reduced personal accomplishment resulting from chronic interpersonal job stressors.^27^ Burnout could also be exacerbated by teachers' concerns about workplace safety, where measures are perceived as inadequately implemented or insufficiently adhered to. Furthermore, burnout and low morale may in turn be associated with lower fidelity of implementation of preventive measures given that staff commitment is commonly a key influence on the implementation of school‐based health interventions.^28^ Bi‐directional causal relations between staff burnout and poor implementation of preventive measures may therefore trigger feedback cycles which hamper prevention efforts in schools. However, to the best of our knowledge, research to date has not explored whether there is any association between implementation of COVID‐19 preventive measures and staff burnout.

Under guidance from England's Department for Education (DfE), primary and secondary schools reopened to all students in September 2020 with multiple preventive measures, including the requirement that individuals who are unwell stay home, use of face‐coverings, hand and respiratory hygiene, enhanced cleaning, and physical distancing.^29^ Face‐coverings in corridors or communal areas were recommended for secondary but not primary schools. Primary school guidance emphasized keeping students in separated small groups (“bubbles”). Secondary school guidance focused on distancing between individuals within larger bubbles. Schools were further required to engage with test and trace systems, exclude infected students and contain outbreaks with advice from local health protection teams. From January 5, 2021, schools were again closed to all except vulnerable or key‐workers' children.^30^ On March 7, 2021 schools re‐opened to all students with revised measures to implement including widespread asymptomatic testing using lateral flow devices and use of face‐coverings in classrooms for secondary‐school students.^31^

Focusing on experiences in the autumn term of 2020 (September‐December 2020), this paper examines the fidelity and feasibility of implementation of COVID‐19 preventive measures in schools, and explores associations between adherence to these measures and staff well‐being, to inform policy on sustainable implementation of such measures and staff mental well‐being.

## METHODS

The COVID‐19 Schools Infection Survey (SIS) was an observational cohort study conducted in state primary and secondary schools across 15 selected local authorities (LAs) in England during the school year 2020/2021. Multi‐stage stratified sampling was used to select schools, oversampling LAs where SARS‐CoV‐2 infection prevalence was higher (top 20% of LAs ranked by population rate of confirmed infection) in September 2020, and oversampling secondary schools within selected LAs. The study aimed to assess the role of schools in SARS‐CoV‐2 transmission and school implementation of preventive measures. SIS involved: repeat SARS‐CoV‐2 antigen and antibody testing; web‐based questionnaire surveys of staff, parents, and older students; and semi‐structured telephone interviews with a sub‐set of schools. Six rounds of data collection were planned over the 2020/2021 school year, with rounds 1 and 2 taking place in November and December 2020, respectively. Further details on the overall study are reported elsewhere.^32^

We considered data from the autumn 2020 school term (rounds 1 and 2) for this analysis where 128 schools provided headteacher or staff questionnaire data.

### Participants and Procedures

As schools enrolled in the study, headteachers or their nominated representatives (usually other school senior leaders) were sent a school‐level headteacher questionnaire and each school was asked to complete one. All staff in participating schools were eligible to enroll in the SIS study. All enrolled staff were initially sent a demographic questionnaire followed by a more extensive questionnaire in mid‐December 2020. Surveys were administered online. Multiple reminder emails were sent and follow‐up telephone calls were made to participants. A telephone helpline was made available to participants to answer queries.

In this paper, we report on school implementation of preventive measures in the autumn term (September‐December) of 2020, when schools were open to all students. This cross‐sectional analysis draws on data from headteacher and staff questionnaires with reference to autumn term 2020, issued to participants in round 1 and 2, completed November 2020‐March 7, 2021. Questionnaires remained open past the end of the autumn term to allow time for more participants to complete them.

### Measures

Fidelity is defined as the degree to which a program is implemented as intended^33^ and in this context refers to the extent to which the suite of preventive measures recommended by the government were implemented in schools. Adherence refers to the extent to which students or teachers implement measures consistent with advice from the school. Feasibility considers the level of challenge associated with implementation and adherence. These measures of implementation were assessed using indicators developed specifically for this project, informed by: previous research conducted in summer 2020;^19^ public documents providing government guidelines on school preventive measures; and discussion with experts.

To understand fidelity and feasibility of implementation, the headteacher questionnaire included a list of potential preventive measures, 46 of which were applicable to primary schools and 47 to secondary schools, and participants were asked whether each of these was implemented at their school and how challenging implementation was. For example, headteachers were asked to indicate whether the measure “All desks face forward” was implemented and if so, whether implementation involved major challenges, some challenges or was easy to implement. The headteacher questionnaire also included questions on bubble sizes, procedures for internal risk assessments and review, and school and headteacher characteristics.

The staff questionnaire included questions on adherence with measures and burnout. Teaching staff were asked about their perception of school‐wide adherence with a subset of 14 measures relating to hygiene and sanitation, social distancing, and face‐covering use among staff and students. They were asked how well, in their experience, each preventive measure was being followed (always, sometimes, rarely, never, not applicable, do not know). A “perceived adherence” score was calculated for each respondent, summing their responses across 10 of these measures classed as “strongly recommended” or “recommended.” For each measure, “always” was scored as 2, “sometimes” as 1, and “never,” “rarely,” “not applicable,” or “do not know” as 0. The potential range of scores was thus 0‐20.

Teacher burnout was assessed using the 22‐item Maslach Burnout Inventory (MBI) Educators Survey. MBI is a validated measure designed to assess 3 dimensions of burnout using subscales focused on emotional exhaustion (9 items), depersonalisation (5 items), and personal accomplishment (8 items).^27^, ^34^ Emotional exhaustion is often considered a key aspect of burnout, while depersonalisation refers to negative, cynical attitudes and feelings toward students and colleagues.^27^ Reduced personal accomplishment refers to a tendency to evaluate oneself negatively and feelings of incompetence and poor achievement in one's work. For each statement of job‐related feelings, participants were asked how often they felt that way, with responses scaled from “never” (0) to “every day” (6). Subscale scores were calculated by summing scores across the relevant items, with higher scores indicating greater burnout for emotional exhaustion and depersonalisation, and lower scores indicating greater burnout in relation to personal accomplishment. In addition to the total scores, a binary measure of high burnout was also created for each subscale, indicated by validated scores of 27 or over for emotional exhaustion, 14 or over for depersonalisation, and 30 or below for personal accomplishment.^35^

### Data Analysis

Data were stored on secure software and exported into the Office for National Statistics (ONS) Data Access Platform. De‐identified datasets were accessed by accredited members of the study team via ONS secure servers. Descriptive tables were generated using Stata MP 16.1 (StataCorp LLC) within ONS's Secure Research Service platform.

**Figure 1:**
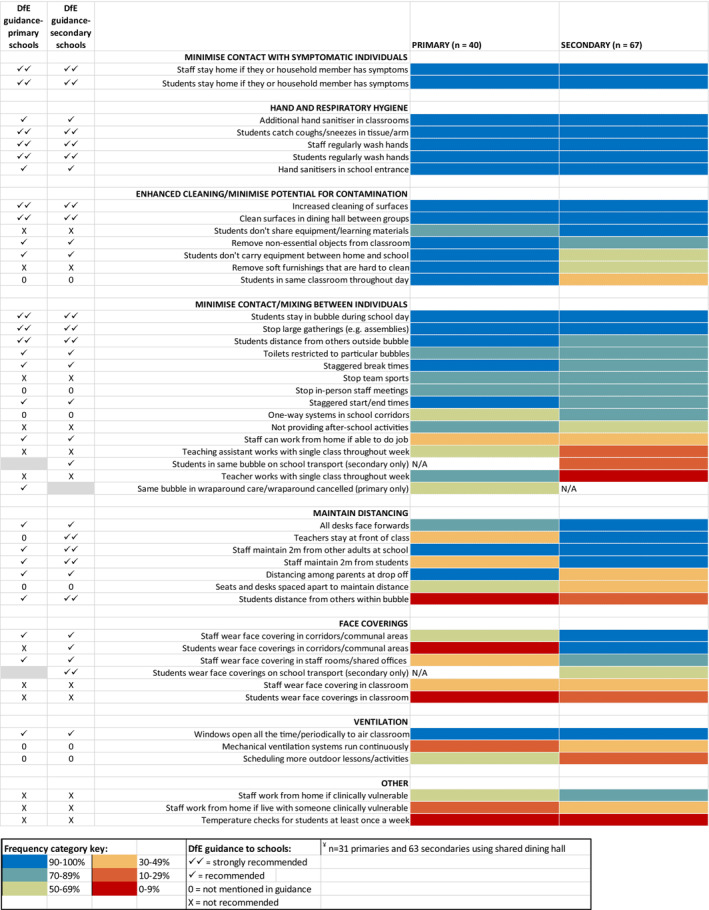
Heatmap Showing Frequency of Implementation of COVID‐19 Measures in Primary and Secondary Schools We present the proportion of schools implementing each COVID‐19 preventive measure, as reported in the headteacher questionnaire. Measures were grouped within 8 domains aligning with government guidance: minimizing contact with symptomatic individuals; hand and respiratory hygiene; enhanced cleaning/minimizing potential for fomite transmission; minimizing mixing between bubbles; maintaining distance between individuals; face‐coverings; ventilation; and other. The research team further categorized measures, separately for primary and secondary schools, as “strongly recommended,” “recommended,” “not mentioned,” or “not recommended” based on their reading of government guidance.

We present the proportion of schools implementing each COVID‐19 preventive measure, as reported in the headteacher questionnaire (Figure 1). Measures were grouped within 8 domains aligning with government guidance.^29^ We also calculate the median number of measures implemented by schools (overall and by level of DfE recommendation), alongside interquartile range (IQR) and range. We further explored whether the number of measures implemented differed according to school‐level socio‐economic disadvantage assessed via student eligibility for free school meals (FSM): up to 20% eligible = low; 21‐30% eligible = medium; >30% eligible = high.

Staff‐reported school‐wide adherence to each preventive measure was assessed among teaching staff working in schools in which headteachers reported that the measure in question was implemented. We present the overall frequency with which staff report that each measure was “always” or “sometimes” followed and the school‐level intra‐cluster correlation coefficient (ICC) for the “always” response.

The analysis of teacher burnout was performed separately for those with leadership positions (ie, headteachers, and senior and middle leaders) and other teaching staff. We present mean scores and standard deviations for each subscale of the MBI, and the percentage of teachers with “high” burnout for each subscale. School‐level ICCs for the mean scores were estimated using an intercept‐only multi‐level linear regression model.

To explore the association between teacher burnout and teacher‐reported school‐wide adherence to COVID‐19 preventive measures, we considered each MBI subscale separately. We conducted multi‐level mixed‐effects linear regression with random intercept for school and the continuous score for each subscale as the dependent variable. In addition to teacher‐reported school‐wide adherence to COVID‐19 prevention measures (calculated among all teaching staff irrespective of whether headteacher reported measure as implemented), independent variables considered in the unadjusted analysis were school type (primary/secondary), percentage of students eligible for FSM (low/medium/high) and government inspection rating (inadequate or requires improvement/good/outstanding). Those variables found to be associated with any of the MBI subscales in these models (p < .1) were then included, alongside teacher's age and gender, in adjusted models for all subscales.

## RESULTS

In total, 128 schools (45 primary and 83 secondary) participated and provided questionnaire data in rounds 1 and 2 of the SIS study. Of these, 107 (84%) schools (40 primary and 67 secondary schools) completed the headteacher questionnaire by March 7, 2021. Among 4566 teaching staff across all 128 schools enrolled in rounds 1 and 2 of the study, 59% (640 primary and 2058 secondary teaching staff) completed the questionnaire by March 7, 2021 and were included in the analysis (Table 1).

**Table 1:** Characteristics of Schools and Respondents to the Headteacher and Staff Questionnaires (From Rounds 1 and 2 of the Study)

	Primary	Secondary
**School‐level characteristics (all schools with headteachers or staff questionnaires completed)** *	n = 45	n = 83
Number of students, median (IQR)	297 (213‐420)	936 (755‐1263)
Number of teachers, median (IQR)	16 (11‐21)	63 (47‐82)
Eligible for FSM band, n (%)		
Low (≤20%)	24 (53)	47 (57)
Medium (21‐30%)	8 (18)	17 (20)
High (>30%)	13 (29)	19 (23)
OFSTED rating, n (%)		
Data not available	6 (13)	17 (20)
Inadequate	0 (0)	5 (6)
Requires improvement	2 (4)	13 (16)
Good	30 (67)	34 (41)
Outstanding	7 (16)	14 (17)
Value added scores, median (IQR) (primary)^†^		
Reading	0.9 (−0.6‐1.8)	—
Writing	0.4 (−1.1‐1.9)	—
Maths	0.7 (−0.5‐2.2)	—
Progress 8 score, median (IQR) (secondary)^‡^	—	0.015 (−0.42 to 0.39)
**Characteristics of participants completing the headteacher questionnaire (prior to March 8, 2021)**	n = 40	n = 67
Headteacher questionnaire ever completed	*44/45 (98%)*	*73/83 (88%)*
Headteacher questionnaire completed prior to March 8, 2021	*40/45 (89%)*	*67/83 (81%)*
Role of participant, n (%)		
Headteacher	33 (83)	37 (55)
Deputy/Assistant head	0 (0)	10 (15)
Other member of senior leadership	6 (15)	11 (16)
Other	1 (3)	9 (13)
Age (years), n (%)		
26‐35	3 (8)	3 (4)
36‐45	11 (28)	29 (43)
46‐55	21 (53)	24 (36)
56+	4 (10)	7 (10)
Prefer not to say	1 (3)	4 (6)
Sex, n (%)		
Male	10 (25)	26 (39)
Female	30 (75)	38 (57)
Prefer not to say	0 (0)	3 (4)
**Characteristics of teaching staff completing staff questionnaire prior to March 8, 2021**	n = 640	n = 2058
Number of teaching staff enrolled in study (Rounds 1 and 2)	*1007*	*3559*
Number of teaching staff ever completing staff questionnaire	*717/1007 (71%)*	*2300/3559 (65%)*
Number of teaching staff completing staff questionnaire prior to 8 March 2021	*640/1007 (64%)*	*2058/3559 (58%)*
Number of teaching staff per school who completed questionnaires (prior to March 8, 2021), median (IQR)	13 (10‐17)	24 (15‐33)
Staff role, n (%)		
Headteacher	32 (5)	23 (1)
Senior leader	53 (8)	176 (9)
Middle leader	62 (10)	613 (30)
Teacher	221 (35)	1012 (49)
Teaching assistant	270 (42)	220 (11)
Other	2 (0.3)	14 (1)
Age (years), mean (SD)	42.4 (10.9)	39.7 (10.1)
Sex, n (%)		
Male	45 (7)	543 (26)
Female	595 (93)	1514 (74)

* School‐level descriptive data were compiled from the following sources: (a) Department for Education. Get information about schools. 2021; Available at: https://www.get‐information‐schools.service.gov.uk/ and (b) Department for Education. Find and compare schools in England. 2021; Available at: https://www.compare‐school‐performance.service.gov.uk/find‐a‐school‐in‐england.

† n = 39 for all primary value added scores.

‡ n = 74; progress 8 score is a value added measure for secondary schools indicating the progress pupils at a school make compared to pupils across England based on results in up to 8 subjects including maths and English.

### School and Participant Characteristics

Secondary schools with a median of 936 students were larger than primary schools that had a median of 297 students (Table 1). Twenty‐nine percent (13/45) of primary and 23% (19/83) of secondary schools in this analysis were in the high FSM band (>30% eligibility), while over half were in the low FSM band. Thirty‐seven (82%) primary and 48 (58%) secondary schools were rated by government inspectors as “good” or “outstanding.”

Most headteacher questionnaires were completed by headteachers themselves (primary: 83%, 33/40; secondary: 55%, 37/67). The median number of teaching staff completing the questionnaire per school was 13 (IQR: 10‐17) for primary and 24 (IQR: 15‐33) for secondary schools. Most participants in both the headteacher and staff questionnaires were women.

### School‐Level Implementation of Preventive Measures

According to the headteacher questionnaire, most primary schools (75%, 30/40) kept students in bubbles of normal class sizes, while bubbles in most secondary schools (72%, 48/67) consisted of year‐groups (Table 2). In about half of both primary and secondary schools, students were seated less than 0.5 m apart in the classroom. Only 1 primary and 3 secondary schools reported that students were seated at least 2 m apart.

**Table 2:** School Implementation of Student “Bubbles” and Spacing Within the Classroom

	Primary, *n* (%)	Secondary, *n* (%)
Items from headteacher questionnaire	n = 40	n = 67
Bubble size		
Smaller than normal class sizes	2 (5)	2 (3)
Normal class sizes	30 (75)	11 (16)
Entire year group*	5 (13)	48 (72)
Other	3 (8)	6 (9)
Distance between student seating in classroom		
<0.5 m apart	20 (50)	32 (48)
>0.5 m and <1 m apart	11 (28)	19 (28)
>1 m and <2 m apart	5 (13)	9 (13)
>2 m apart	1 (3)	3 (4)
Other	3 (8)	3 (4)
Do not know/prefer not to say	0 (0)	1 (1)
Items from staff questionnaire	n = 518	n = 1703
At some times in the day students might mix with students in other bubbles^†^	121/466 (26)	780/1496 (52)
Times when students mix (among those reporting mixing)	n = 121	n = 780
Lunch	64 (53)	345 (44)
Break	47 (39)	355 (46)
Sports	5 (4)	67 (9)
Other	58 (48)	543 (70)

* Secondary school year group size: median 171 students, IQR 144‐215, range 58‐300.

† Fifty‐two excluded from primary school denominator for not providing yes/no response (3 Prefer not to say, 23 Do not know, 26 N/A), 207 excluded from secondary school denominator (7 Prefer not to say, 170 Do not know, 30 N/A).

The frequencies with which primary and secondary schools reported implementing specific COVID‐19 preventive measures (headteacher questionnaire) are presented in Figure 1. Measures to minimize contact with symptomatic individuals and measures relating to hand/respiratory hygiene were implemented by almost all schools. Enhanced cleaning was also adopted by almost all, though primary schools were more likely to implement additional measures to limit fomite transmission. The most commonly adopted measures to minimize contacts between individuals included keeping students in bubbles and stopping large gatherings. Overall, implementation of measures to prevent mixing between bubbles was slightly lower in secondary than primary schools.

However, measures to maintain distance between individuals were more widely implemented in secondary than primary schools (eg, desks faced forwards, teachers stayed at the front of the class, and staff maintained at least 2 m distance from students). Primary schools were unlikely to implement distancing measures (these measures were not emphasized in the guidance for primary schools). Under a quarter of secondary schools reported implementing distancing between students within the same bubble.

All secondary‐school headteachers reported that staff and students wore face‐coverings in corridors and other communal areas. Forty‐seven percent of primary and 33% of secondary schools reported that teachers were required to use face‐coverings in classrooms. Policy requiring use of face‐coverings by students in the classroom was much less frequent, reported by under one‐fifth of secondary schools and 1 primary school.

All primary and most secondary‐schools opened windows to ventilate classrooms. Mechanical ventilation systems were present in very few primary and 36% of secondary schools.

The median number of measures implemented by primary schools was 33 (IQR: 31‐35.5, range 23‐41), and by secondary schools was 32 (IQR: 30‐35, range 22‐40). Almost all primary schools (93%, 37/40) reported implementing all 10 measures strongly recommended in government guidance. Of the 67 secondary schools, 7 (10%) implemented all 15 measures strongly recommended in government guidance, although 62 (93%) reported implementing at least 12. The strongly recommended measure least likely to be implemented was maintaining distance between students within bubbles (26%). No differences were noted regarding number of measures implemented by low‐, medium‐, and high‐FSM schools.

Most headteachers reported conducting regular internal reviews of COVID‐related policies and measures within their schools: Of the 40 primary and 67 secondary schools, 38% of primary and 30% of secondary schools did these reviews once to twice per month, while 33% of primary and 31% of secondary schools did them once or twice per week.

### Challenges Associated With Implementing Preventive Measures

Headteachers reported that most measures presented challenges to schools (Supporting Information). Regular handwashing for staff and installing hand sanitisers at school entrances were considered easy to implement. Other measures considered easy to implement by a majority of headteachers were: (in primary) keeping students in the same classroom all day, stopping large gatherings and team sports, and not providing after‐school activities; (in secondary) staff wearing face‐coverings in corridors and staff rooms. Secondary schools tended to report more challenges than primary schools implementing measures to minimize mixing between bubbles and potential for contamination. For example, keeping students in the same classroom all day was considered majorly challenging in 41% (12/29) of secondary, and easy to implement in 70% (26/37) of primary schools, reporting implementing this measure.

### School‐Wide Adherence With Preventive Measures

Levels of staff‐reported school‐wide adherence with prevention measures varied by measure and between primary and secondary schools (Table 3). High levels of adherence were reported for measures such as staff wearing face‐coverings in corridors and communal areas, staff regularly washing their hands, secondary schools having desks facing forwards, and increased cleaning of surfaces and student handwashing in primary schools.

**Table 3:** School‐Wide Adherence With COVID‐19 Preventive Measures Reported by (a) Primary School Teaching Staff and (b) Secondary School Teaching Staff

Preventive Measure	Number of Schools	Total Respondents	“Do Not Know” Response, n (%)	No. Providing Response	Never/ Rarely/NA, n (%)	Sometimes, n (%)	Always, n (%)	School‐Level ICC for “Always” Response, ICC (95%CI)
(a) Primary school teaching staff								
Staff wear face covering in classroom	19	223	2 (1)	221	40 (18)	79 (36)	102 (46)	0.451 (0.244‐0.676)**
Staff wear face covering in corridors/communal areas	25	364	2 (1)	362	19 (5)	81 (22)	262 (72)	0.407 (0.228‐0.614)**
Staff wear face covering in staffroom/shared office	19	263	6 (2)	257	21 (8)	89 (35)	147 (57)	0.138 (0.043‐0.362)*
Staff maintain 2 m distance from students	16	210	7 (3)	203	69 (34)	99 (49)	35 (17)	0.062 (0.007‐0.396)
Staff maintain 2 m distance from others adults in school	38	503	10 (2)	493	29 (6)	237 (48)	227 (46)	0.172 (0.087‐0.312)**
Staff regularly wash/sanitize hands	40	517	25 (5)	492	0 (0)	44 (9)	448 (91)	0.000 (0.000‐0.000)
Students wear face covering in classroom	1	9	0 (0)	9	8 (89)	0 (0)	1 (11)	0.000 (0.000‐0.000)
Students regularly wash/sanitize hands	39	509	4 (1)	505	3 (1)	55 (11)	447 (89)	0.075 (0.011‐0.365)
Students catch coughs/sneezes in tissue/arm	40	517	12 (2)	505	81 (16)	301 (60)	123 (24)	0.014 (0.000‐0.418)
Seats and desks spaced apart	24	279	2 (1)	277	91 (33)	67 (24)	119 (43)	0.209 (0.090‐0.411)**
All desks face forward	34	433	1 (0.2)	432	81 (19)	83 (19)	268 (62)	0.107 (0.040‐0.254)**
Increased cleaning of surfaces	40	517	6 (1)	511	29 (6)	104 (20)	378 (74)	0.280 (0.150‐0.462)**
Students do not share equipment in class	31	397	4 (1)	393	109 (28)	139 (35)	145 (37)	0.283 (0.156‐0.457)**
Students do not carry equipment between home and school	36	435	8 (2)	427	96 (22)	136 (32)	195 (46)	0.064 (0.018‐0.209)*
(b) Secondary school teaching staff								
Staff wear face covering in classroom	22	490	12 (2)	478	115 (24)	203 (42)	160 (33)	0.080 (0.028‐0.210)**
Staff wear face covering in corridors/communal areas	67	1471	1 (0.1)	1470	6 (0.4)	174 (12)	1290 (88)	0.137 (0.073‐0.244)**
Staff wear face covering in staffroom/shared office	47	981	20 (2)	961	122 (13)	530 (55)	309 (32)	0.144 (0.079‐0.248)**
Staff maintain 2 m distance from students	61	1330	15 (1)	1315	151 (11)	883 (67)	281 (21)	0.051 (0.020‐0.122)*
Staff maintain 2 m distance from others adults in school	63	1362	16 (1)	1346	69 (5)	875 (65)	402 (30)	0.029 (0.009‐0.088)*
Staff regularly wash/sanitize hands	63	1364	163 (12)	1201	13 (1)	333 (28)	855 (71)	0.050 (0.020‐0.119)*
Students wear face covering in classroom	12	265	3 (1)	262	152 (58)	92 (35)	18 (7)	0.145 (0.019‐0.599)
Students regularly wash/sanitize hands	63	1402	135 (10)	1267	154 (12)	730 (58)	383 (30)	0.231 (0.151‐0.337)**
Students catch coughs/sneezes in tissue/arm	64	1402	189 (13)	1213	262 (22)	781 (64)	170 (14)	0.081 (0.033‐0.188)**
Seats and desks spaced apart	28	537	5 (1)	532	260 (49)	147 (28)	125 (24)	0.000 (0.000‐0.000)
All desks face forward	66	1449	6 (0.4)	1443	40 (3)	288 (20)	1115 (77)	0.143 (0.084‐0.231)**
Increased cleaning of surfaces	66	1455	53 (4)	1402	110 (8)	457 (33)	835 (60)	0.172 (0.108‐0.263)**
Students do not share equipment in class	63	1348	31 (2)	1317	241 (18)	692 (53)	384 (29)	0.145 (0.087‐0.230)**
Students do not carry equipment between home and school	39	841	62 (7)	779	350 (45)	270 (35)	159 (20)	0.073 (0.028‐0.176)**

* p < .05;

** p = <.001.

ICC, Intra‐Cluster Correlation Coefficient.

Staff‐reported school‐wide adherence to each preventive measure was assessed among teaching staff working in schools in which headteachers reported that the measure in question was implemented. We present the overall frequency with which staff report that each measure was “always” or “sometimes” followed and the school‐level ICC for the “always” response.

Many measures were frequently reported as “sometimes” followed. These included staff wearing face‐coverings in classrooms and staffrooms, staff distancing from students and other adults, student catching coughs and sneezes in a tissue, and students not sharing equipment in class or carrying equipment between school and home.

Secondary‐school staff reported higher adherence than primary schools for some measures, such as staff distancing from students, desks facing forwards and students not sharing equipment. However, for most measures, adherence was reported as worse in secondary than primary schools. More staff in secondary schools also answered “do not know” regarding adherence with measures relating to staff and student behaviors. Across primary and secondary schools, ICCs indicated that staff perception of adherence to measures related to school environments or mandated observable behaviors (such as face‐covering use and increased cleaning of surfaces) tended to exhibit more within‐school clustering than measures related to individual behavior (such as maintaining distancing, students catching coughs/sneezes and students carrying equipment between school and home).

### Teacher Burnout and Well‐Being

Over half those in leadership positions and 42% (517/1234) of other teaching staff suffered high burnout regarding emotional exhaustion (Table 4a). Six percent (40/629) of leaders and 15% (n = 180/1226) of other teaching staff reported high burnout regarding personal accomplishment. Few (3%) reported high burnout regarding depersonalisation. Mean scores indicate higher burnout among leaders than other teachers regarding emotional exhaustion and depersonalisation, and lower burnout among leaders regarding personal accomplishment. Lower teacher‐reported school‐wide adherence with measures was consistently associated across all 3 domains with higher burnout among leaders and other teaching staff (Table 4b). School‐type was also associated with teacher burnout, with secondary‐school leaders and other teachers more likely than those in primary to experience greater emotional exhaustion, greater depersonalisation and lesser personal accomplishment. Teachers in more deprived (high‐FSM) schools were less likely to experience emotional exhaustion and depersonalisation than teachers at schools with low FSM.

**Table 4:** (a) Descriptive Data on 3 Domains of Teacher Burnout Assessed Using the Maslach Burnout Inventory, Disaggregated by Leadership Roles/Other Teaching Staff.^†^ (b) Multivariate Regression Beta Coefficients^‡^ (95% CI) for the Association Between 3 Domains of Teacher Burnout and Teacher Perception of School‐Wide Compliance With COVID‐19 Preventive Measures

	Heads/Senior Leaders/Middle Leaders			Other Teaching Staff		
	Emotionalexhaustion, n = 631 (115 schools)	Depersonalisation, n = 628 (116 schools)	Personal accomplishment, n = 629 (116 schools)	Emotional exhaustion, n = 1234 (127 schools)	Depersonalisation, n = 1239 (127 schools)	Personal accomplishment, n = 1226 (127 schools)
(a)						
Mean (SD)	26.5 (11.7)	3.5 (4.1)	39.1 (5.6)	23.9 (12.9)	3.2 (4.1)	37.8 (6.6)
School‐level ICC	0.000 (0.000‐0.000)	0.050 (0.018‐0.134)*	0.000 (0.000‐0.000)	0.070 (0.038‐0.126)**	0.079 (0.046‐0.135)**	0.021 (0.005‐0.094)
n (%) with high burnout	324 (51)	19 (3)	40 (6)	517 (42)	36 (3)	180 (15)
(b)						
Teacher reported compliance with COVID‐19 prevention measures (linear term), b (95% CI)	−0.78 (−1.07 to −0.49)**	−0.27 (−0.37 to −0.17)**	0.34 (0.20‐0.48)**	−0.96 (−1.16 to −0.75)**	−0.21 (−0.27 to −0.15)**	0.38 (0.27‐0.49)**
Other school‐level variables, b (95%CI)						
School type						
Primary	—	—	—	—	—	—
Secondary	0.68 (−1.97 to 3.32)	1.05 (0.10‐2.00)*	−1.46 (−2.75 to −0.18)*	3.23 (1.34‐5.13)**	1.48 (0.90‐2.05)**	−1.31 (−2.19 to −0.44)*
Percentage of students on FSM						
Low	—	—	—	—	—	—
Medium	−0.92 (−3.20 to 1.36)	−0.15 (−1.02 to 0.72)	0.65 (−0.46 to 1.76)	−0.05 (−2.26 to 2.16)	0.15 (−0.52 to 0.82)	−0.52 (−1.52 to 0.47)
High	0.50 (−1.88 to 2.88)	−0.20 (−1.08 to 0.68)	0.22 (−0.92 to 1.35)	−3.37 (−5.49 to −1.25)*	−0.67 (−1.32 to −0.03)*	0.21 (−0.76 to 1.19)
ICC (95%CI)	0.000 (0.000‐0.000)	0.041 (0.012‐0.130)*	0.000 (0.000‐0.000)	0.043 (0.019‐0.094)**	0.036 (0.014‐0.087)*	0.007 (0.000‐0.271)

* p < .05;

** p < .1.

ICC, Intra‐Cluster Correlation Coefficient.

† The 3 dimensions of burnout were emotional exhaustion, depersonalisation, and personal accomplishment, with higher scores indicating greater burnout for emotional exhaustion and depersonalisation, and lower scores indicating greater burnout in relation to personal accomplishment. In addition to the total scores, a binary measure of high burnout has been created for each subscale, indicated by validated scores of 27 or over for emotional exhaustion, 14 or over for depersonalisation, and 30 or below for personal accomplishment.

‡ Beta coefficients adjusted for all variables in this table, and teacher's age and gender; estimated using a multi‐level mixed‐effects linear regression model with random intercept for school.

## DISCUSSION

Despite concerns about the feasibility of implementing preventive measures in schools^36^, ^37^ and notwithstanding challenges reported, most schools in our study implemented many preventive measures. Fidelity of implementation varied across schools, likely due to challenges interpreting guidance received, physical constraints of school environments and resources.^19^, ^22^, ^38^ Though not all secondary schools in our study implemented all strongly recommended measures, many schools did more than advised in national guidance at the time. For example, as reported in other studies,^39^ some primary schools implemented use of face‐coverings.

Secondary‐school staff were more likely to report poorer adherence to many measures related to staff and student behavior. This is likely indicative of the challenges of implementing measures in large institutions. A study in Germany postulated more flexible adaptation in primary schools and a more dismissive attitude among older students toward regulations on social behavior as possible reasons for better implementation of preventive measures in primary than secondary schools.^39^ Furthermore, some guidance for secondary schools may have been unrealistic, making poor adherence likely, as implementation theory would predict.^40^ For example, less than a third of secondary schools reported implementing distancing for students within their bubbles, despite this being strongly recommended in the guidance. On the other hand, the older age of students in secondary schools may facilitate adherence to other distancing measures such as staff distancing from students and desks facing forwards.

We also found that secondary‐school teachers were more likely to experience burnout and teachers working in schools with more economically disadvantaged students were less likely to suffer from emotional exhaustion and depersonalisation. Other research looking into why teachers stay in challenging schools found that wanting to make a difference, a strong sense of social responsibility and rewarding relationships with students, even where challenging, were key factors,^41^, ^42^ and it is possible that fulfillment of these ideals may act as protective factors against burnout among teachers in these schools. However, this analysis is focused on the association between reported adherence and burnout, and is not a comprehensive assessment of factors influencing teacher burnout; these findings, therefore, must be interpreted accordingly.

The high levels of burnout among teachers, and especially school leaders, in this study are consistent with anecdotal reports of the challenges teachers have faced and the negative impacts on their well‐being,^22^ although we have no comparison to levels of burnout before the pandemic. We found a consistent association between burnout and lower teacher‐perceived school‐wide adherence with COVID‐19 measures. Though the cross‐sectional nature of this analysis precludes inference about causality, it is plausible both that school environments in which preventive measures are poorly adhered to may contribute to teacher burnout and that teacher burnout could undermine implementation of COVID‐19 measures. Finally, as adherence with preventive measures was a subjective measure based on teachers' perceptions, it is possible that teachers with higher burnout were more likely to perceive poor adherence with preventive measures.

### Limitations

Of 289 schools initially approached for the SIS study, 128 schools participated and provided questionnaire data in rounds 1 and 2. Although response rates to the headteacher questionnaire were high, with completion by 107 of the 128 participating schools, it is possible schools that did not sign‐up for the study or where headteachers did not respond differed in important ways from those in which headteacher questionnaires were completed. They may, for example, have had less rigorous protocols around COVID‐19 prevention than those included in this analysis. Response rates were also lower than expected for the staff questionnaire, again leading to a potentially non‐representative sample of teaching staff and biased estimates for adherence to measures and teacher burnout. Participants were allowed to complete the questionnaires after the end of the autumn term to allow sufficient time to complete the questionnaire as they were made available to participants only toward the end of the autumn term. We do not believe asking participants to reflect on the previous term is likely to have been too problematic given that schools were closed to most students during the following term. Oversampling of LAs where SARS‐CoV‐2 infection prevalence was high in Sep 2020 was done to facilitate transmission studies; this may limit the ability to generalize findings for England. However, our sample of schools from 15 different LAs and varying Covid‐19 prevalence over the year makes it unlikely that implementation practices in our schools differed widely from others in England. Finally, the cross‐sectional nature of this analysis does not allow us to fully explore whether poor adherence with preventive measures resulted in greater teacher burnout or vice versa.

## IMPLICATIONS FOR SCHOOL HEALTH POLICY AND PRACTICE

The large number of new policies and preventive measures that schools had to innovate and implement, the challenge they have presented, and the task of regularly reviewing policies and ensuring adherence to them have increased the workload of school leaders and teachers. Findings suggest the need for clear technical guidance based on consultation with schools.^19^, ^22^ However, the finding that a similar number of preventive measures were implemented at school‐level irrespective of socio‐economic advantage of schools, reassuringly suggests an equitable provision of guidance.

Our findings support the case for investing in teacher mental health and well‐being. This investment is essential in itself to decrease teacher burnout and is likely also key to ensuring sustainability of effective implementation of preventive measures. Furthermore, our findings may also suggest that supporting better implementation of and adherence to preventive measures may decrease teacher burnout. The wider teacher burnout literature, which suggests that classroom environments and discipline, lack of clear rules, and ineffective implementation and enforcement of rules may impact burnout, supports these findings.^43^, ^44^ Our findings also suggest the importance of ensuring school working environments feel safe for staff. Other research recommends supporting teachers to feel autonomous, competent and connected with colleagues, students and their families for their well‐being^37^ and investment in training programs for stress reduction.^45^, ^46^, ^47^

Measures that may be worth considering for ongoing implementation, if needed, as schools transition to normal functioning are those that were relatively less challenging to implement and secured higher adherence such as, face‐coverings for staff, frequent handwashing, enhanced cleaning, and (secondary schools) forward‐facing desks.

Further research is required to explore fidelity and feasibility of measures introduced after autumn term 2020 such as regular asymptomatic testing, ongoing maintenance of those COVID‐19 preventive measures that continue to be required of schools, as well as to assess the mental health of staff and students as schools gradually return to normal functioning.

### Human Subjects Approval Statement

This study received approval from research ethics committees at the UK Health Security Agency (ref: NR0237) and the London School of Hygiene and Tropical Medicine (ref: 22657). Participants provided consent on the online portal before beginning the survey questionnaire.

### Conflict of Interest

All authors have no conflicts of interest to disclose.

### Disclaimer

This work contains statistical data from ONS which is Crown Copyright. The use of the ONS statistical data in this work does not imply the endorsement of the ONS in relation to the interpretation or analysis of the statistical data. This work uses research datasets which may not exactly reproduce National Statistics aggregates.

## Supporting information


**Data S1.** Challenges implementing COVID‐19 measures in primary schools.
**Data S2**. Challenges implementing COVID‐19 measures in secondary schools.

## References

[1] World Health Organization . WHO coronavirus (COVID‐19) dashboard. 2021. Available at: https://covid19.who.int/. Accessed November 8, 2021.

[2] Götzinger F , Santiago‐García B , Noguera‐Julián A , et al. COVID‐19 in children and adolescents in Europe: a multinational, multicentre cohort study. Lancet Child Adolesc Health. 2020;4:653‐661. 10.1016/s2352-4642(20)30177-2. 32593339 PMC7316447

[3] Swann OV , Holden KA , Turtle L , et al. Clinical characteristics of children and young people admitted to hospital with covid‐19 in United Kingdom: prospective multicentre observational cohort study. BMJ. 2020;370:m3249. 10.1136/bmj.m3249. 32960186 PMC7488201

[4] Viner RM , Mytton OT , Bonell C , et al. Susceptibility to SARS‐CoV‐2 infection among children and adolescents compared with adults: a systematic review and meta‐analysis. JAMA Pediatr. 2020;175:143‐156. 10.1001/jamapediatrics.2020.4573. PMC7519436 32975552

[5] Zimmermann P , Curtis N . COVID‐19 in children, pregnancy and neonates: a review of epidemiologic and clinical features. Pediatr Infect Dis J. 2020;39:469‐477. 10.1097/inf.0000000000002700. 32398569 PMC7363381

[6] Ladhani SN , Amin‐Chowdhury Z , Davies HG , et al. COVID‐19 in children: analysis of the first pandemic peak in England. Arch Dis Child. 2020;105:1180‐1185. 10.1136/archdischild-2020-320042. 32796006 PMC7431771

[7] UNESCO . Global monitoring of school closures caused by COVID‐19. 2021. Available at: https://en.unesco.org/covid19/educationresponse. Accessed July 15, 2021.

[8] Viner R , Russell S , Saulle R , et al. Impacts of school closures on physical and mental health of children and young people: a systematic review. medRxiv. 2021:2021.2002.2010.21251526. 10.1101/2021.02.10.21251526.

[9] Bignardi G , Dalmaijer ES , Anwyl‐Irvine AL , et al. Longitudinal increases in childhood depression symptoms during the COVID‐19 lockdown. Arch Dis Child. 2021;106:791‐797. 10.1136/archdischild-2020-320372. PMC7733224 33298552

[10] Hoffman JA , Miller EA . Addressing the consequences of school closure due to COVID‐19 on children's physical and mental well‐being. World Med Health Policy. 2020;12:300‐310. 10.1002/wmh3.365. 32904951 PMC7461306

[11] Kneale D , O'Mara‐Eves A , Rees R , Thomas J . School closure in response to epidemic outbreaks: systems‐based logic model of downstream impacts. F1000Res. 2020;9:352. 10.12688/f1000research.23631.1. 32864104 PMC7445561

[12] Tomasik MJ , Helbling LA , Moser U . Educational gains of in‐person vs. distance learning in primary and secondary schools: a natural experiment during the COVID‐19 pandemic school closures in Switzerland. Int J Psychol. 2021;56:566‐576. 10.1002/ijop.12728. 33236341 PMC7753520

[13] Cullinane C , Montacute R . COVID‐19 and social mobility impact brief #1: school closures. The Sutton Trust; 2020. Available at: https://www.suttontrust.com/wp‐content/uploads/2021/01/School‐Shutdown‐Covid‐19.pdf. Accessed November 8, 2021.

[14] Bayham J , Fenichel EP . Impact of school closures for COVID‐19 on the US health‐care workforce and net mortality: a modelling study. Lancet Public Health. 2020;5:E271‐E278. 10.1016/s2468-2667(20)30082-7. 32251626 PMC7270508

[15] Baron EJ , Goldstein EG , Wallace CT . Suffering in silence: how COVID‐19 school closures inhibit the reporting of child maltreatment. J Public Econ. 2020;190:104258. 10.1016/j.jpubeco.2020.104258. 32863462 PMC7441889

[16] Guthrie BL , Tordoff DM , Meisner J , et al. Summary of school reopening models and implementation approaches during the COVID 19 pandemic. 2020. Available at: https://globalhealth.washington.edu/sites/default/files/COVID‐19%20Schools%20Summary%20%28updated%29.pdf. Accessed July 15, 2021.

[17] Hoffman KS , Torres MB , Wotipka CM . Cross‐national variation in school reopening measures during the COVID‐19 pandemic. AERA Open. 2021;7. 10.1177/23328584211010180.

[18] Melnick H , Darling‐Hammond L . Reopening Schools in the Context of COVID‐19: Health and Safety Guidelines from Other Countries (Policy Brief). Palo Alto, CA: Learning Policy Institute; 2020. Available at: https://learningpolicyinstitute.org/product/reopening‐schools‐covid‐19‐brief. Accessed November 8, 2021.

[19] Sundaram N , Bonell C , Ladhani S , et al. Implementation of preventive measures to prevent COVID‐19: a national study of English primary schools in summer 2020. Health Educ Res. 2021;36:272‐285. 10.1093/her/cyab016. 33860299 PMC8083280

[20] Krishnaratne S , Pfadenhauer LM , Coenen M , et al. Measures implemented in the school setting to contain the COVID‐19 pandemic: a rapid scoping review. Cochrane Database Syst Rev. 2020;12:CD013812. 10.1002/14651858.CD013812. 33331665 PMC9206727

[21] Lessler J , Grabowski MK , Grantz KH , et al. Household COVID‐19 risk and in‐person schooling. Science. 2021;372:1092‐1097. 10.1126/science.abh2939. 33927057 PMC8168618

[22] Education Support . Covid‐19 and the classroom: working in education during the coronavirus pandemic. The impact on education professionals' mental health and wellbeing. London; 2020. Available at: https://www.educationsupport.org.uk/sites/default/files/resources/covid‐19_and_the_classroom.pdf. Accessed July 15, 2021.

[23] Nearchou F , Flinn C , Niland R , Subramaniam SS , Hennessy E . Exploring the impact of COVID‐19 on mental health outcomes in children and adolescents: a systematic review. Int J Environ Res Public Health. 2020;17:8479. 10.3390/ijerph17228479. PMC7698263 33207689

[24] Hakanen JJ , Bakker AB , Schaufeli WB . Burnout and work engagement among teachers. J Sch Psychol. 2006;43:495‐513. 10.1016/j.jsp.2005.11.001.

[25] Saloviita T , Pakarinen E . Teacher burnout explained: Teacher‐, student‐, and organisation‐level variables. Teach Teach Educ. 2021;97:103221. 10.1016/j.tate.2020.103221.

[26] Naghieh A , Montgomery P , Bonell CP , Thompson M , Aber JL . Organisational interventions for improving wellbeing and reducing work‐related stress in teachers. Cochrane Database Syst Rev. 2015:Cd010306. 10.1002/14651858.CD010306.pub2. 25851427 PMC10993096

[27] Maslach C , Jackson SE . The measurement of experienced burnout. J Organ Behav. 1981;2:99‐113.

[28] Herlitz L , MacIntyre H , Osborn T , Bonell C . The sustainability of public health interventions in schools: a systematic review. Implement Sci. 2020;15:4. 10.1186/s13012-019-0961-8. 31906983 PMC6945701

[29] Department for Education . Guidance for full opening: schools. Updated 28 August 2020, Available at: https://webarchive.nationalarchives.gov.uk/ukgwa/20200831003215/https:/www.gov.uk/government/publications/actions‐for‐schools‐during‐the‐coronavirus‐outbreak/guidance‐for‐full‐opening‐schools. Accessed August 25, 2021.

[30] Cabinet Office—Government of the United Kingdom . National lockdown: stay at home. January 4, 2021. Available at: https://webarchive.nationalarchives.gov.uk/ukgwa/20210107164440/https:/www.gov.uk/guidance/national‐lockdown‐stay‐at‐home. Accessed August 25, 2021.

[31] Department for Education . Schools coronavirus (COVID‐19) operational guidance. February 2021. Available at: https://webarchive.nationalarchives.gov.uk/ukgwa/20210303165729/https:/www.gov.uk/government/publications/actions‐for‐schools‐during‐the‐coronavirus‐outbreak?priority‐taxon=b350e61d‐1db9‐4cc2‐bb44‐fab02882ac25. Accessed August 25, 2021.

[32] Halliday KE , Nguipdop‐Djomo P , Oswald WE , et al. The COVID‐19 schools infection survey in England: protocol and participant profile for a prospective, observational cohort study. JMIR Res Protoc. 2022;11:e34075. https://doi.org/10.2196/34075. 10.2196/34075 PMC9651002 35635843

[33] Dusenbury L , Brannigan R , Falco M , Hansen WB . A review of research on fidelity of implementation: implications for drug abuse prevention in school settings. Health Educ Res. 2003;18:237‐256. 10.1093/her/18.2.237. 12729182

[34] Maslach C , Jackson SE , Leiter MP . Maslach Burnout Inventory. In: Zalaquett CP, Wood RJ, eds, Evaluating stress: A book of resources, 3rd ed. Lanham: Scarecrow Education; 1997:191‐218.

[35] Maslach C , Jackson SE , Leiter MP . Maslach Burnout Inventory Manual. 3rd ed. Menlo Park, CA: Mindgarden; 1996.

[36] Smith LE , Woodland L , Amlôt R , Rubin A , Rubin GJ . A cross‐sectional survey of parental perceptions of COVID‐19 related hygiene measures within schools and adherence to social distancing in journeys to and from school. BMJ Paediatr Open. 2020;4:e000825. 10.1136/bmjpo-2020-000825. PMC7482100 34192178

[37] Kim LE , Leary R , Asbury K . Teachers' narratives during COVID‐19 partial school reopenings: an exploratory study. Educ Res. 2021;63:244‐260.

[38] Lorenc A , Kesten JM , Kidger J , Langford R , Horwood J . Reducing COVID‐19 risk in schools: a qualitative examination of secondary school staff and family views and concerns in the South West of England. BMJ Paediatr Open. 2021;5:e000987. 10.1101/2020.10.25.20216937. PMC7948157 34192194

[39] Hommes F , van Loon W , Thielecke M , et al. SARS‐CoV‐2 infection, risk perception, behaviour and preventive measures at schools in Berlin, Germany, during the early post‐lockdown phase: a cross‐sectional study. Int J Environ Res Public Health. 2021;18:2739. 10.3390/ijerph18052739. 33800392 PMC7967466

[40] May C . Towards a general theory of implementation. Implement Sci. 2013;8:18. 10.1186/1748-5908-8-18. 23406398 PMC3602092

[41] Arthur L. What makes teachers decide to stay in challenging schools? 2020. Available at: https://www.thebritishacademy.ac.uk/blog/summer‐showcase‐2020‐what‐makes‐teachers‐decide‐stay‐challenging‐schools/. Accessed September 29, 2021.

[42] CooperGibson Research . Factors affecting teacher retention: qualitative investigation. Report No. DFE‐RR784, 2018. Available at: https://assets.publishing.service.gov.uk/government/uploads/system/uploads/attachment_data/file/686947/Factors_affecting_teacher_retention_‐_qualitative_investigation.pdf. Accessed September 29, 2021.

[43] El Helou M , Nabhani M , Bahous R . Teachers' views on causes leading to their burnout. Sch Leadersh Manag. 2016;36:551‐567. 10.1080/13632434.2016.1247051.

[44] McLain BP . Environmental support and music teacher burnout. Bull Counc Res Music Educ. 2005;164:71‐84.

[45] Zadok‐Gurman T , Jakobovich R , Dvash E , et al. Effect of inquiry‐based stress reduction (IBSR) intervention on well‐being, resilience and burnout of teachers during the COVID‐19 pandemic. Int J Environ Res Public Health. 2021;18:3689. 33916258 10.3390/ijerph18073689 PMC8037267

[46] Schnaider‐Levi L , Ganz AB , Zafrani K , et al. The effect of inquiry‐based stress reduction on teacher burnout: a controlled trial. Brain Sci. 2020;10:468. 10.3390/brainsci10070468. 32708055 PMC7407507

[47] Pozo‐Rico T , Gilar‐Corbí R , Izquierdo A , Castejón JL . Teacher training can make a difference: tools to overcome the impact of COVID‐19 on primary schools. An experimental study. Int J Environ Res Public Health. 2020;17:8633. 10.3390/ijerph17228633. 33233750 PMC7699930

