# Supplementary material for: Implementation of COVID‐19 Preventive Measures and Staff Well‐Being in a Sample of English Schools 2020‐2021
Source: J Sch Health. 2022 Nov 30:10.1111/josh.13264. Online ahead of print. doi: 10.1111/josh.13264 (PMC9877738; doi:10.1111/josh.13264)
Supplement: Supplementary file 1 — Data S1. Challenges implementing COVID‐19 measures in primary schools. Data S2. Challenges implementing COVID‐19 measures in secondary schools. [file JOSH-9999-0-s001.docx]

**Implementation of Covid-19 Preventive Measures and Staff Well-Being in a Sample of English Schools 2020-2021**

**Supplementary material.** Challenges implementing COVID-19 measures in primary schools^±^

^±^Frequency of challenge reported with implementing preventive measures among schools where the measure was reported to be implemented

Measures have been ordered by ease of implementation within each domain

**Supplementary material.** Challenges implementing COVID-19 measures in secondary schools^±^

^±^Frequency of challenge reported with implementing preventive measures among schools where the measure was reported to be implemented

Measures have been ordered by ease of implementation within each domain

Among schools implementing each measure, we present the proportion in which headteachers reported major or some challenges implementing the measure.
